# The modular curriculum of medicine at the Charité Berlin – a project report based on an across-semester student evaluation

**DOI:** 10.3205/zma001262

**Published:** 2019-10-15

**Authors:** Tanja Hitzblech, Asja Maaz, Torsten Rollinger, Sabine Ludwig, Susanne Dettmer, Wiebke Wurl, Yadira Roa-Romero, Raphael Raspe, Mandy Petzold, Jan Breckwoldt, Harm Peters

**Affiliations:** 1 Charité – Universitätsmedizin Berlin, Prodekanat für Studium und Lehre, Dieter Scheffner Fachzentrum für medizinische Hochschullehre und Ausbildungsforschung, Berlin, Germany; 2 Charité – Universitätsmedizin Berlin, Prodekanat für Studium und Lehre, Team Spezielle Lehrformate, Berlin, Germany; 3 Charité – Universitätsmedizin Berlin, Prodekanat für Studium und Lehre, Team Qualitätssicherung und Evaluation, Berlin, Germany; 4 Charité – Universitätsmedizin Berlin, Institut für Medizinische Soziologie und Rehabilitationswissenschaft, Berlin, Germany; 5 Charité – Universitätsmedizin Berlin, Fachschaftsinitiative Medizin, Berlin, Germany

**Keywords:** undergraduate medical education, curriculum development, competency-based education, outcome-orientation, student evaluation

## Abstract

**Aim:** The introduction of a reform clause into the German licensing laws for medical doctors has enabled German faculties to pilot alternative designs for medical degree programmes. The aim of this project report is to outline the curricular features of the modular curriculum of medicine (MCM) at the Charité and to assess the results of its implementation based on a student evaluation across semesters.

**Project outline: **The MCM was planned and implemented in a competency- and outcome-based manner from 2010-2016 in a faculty-wide process. The curriculum is characterised by a modular structure, longitudinal teaching formats and the integration of basic and clinical science. In the winter semester 2017, evaluations by students in semesters 1-10 were carried out. The results were analysed descriptively, and the coverage of overarching learning outcomes was compared to the results of a survey carried out amongst students on the traditional regular curriculum of medicine track in 2016.

**Results: **A total of 1,047 students participated in the across-semester evaluation (return rate 35%). A high percentage of the respondents positively rated the achieved curricular integration and longitudinal teaching formats. The majority of the respondents agreed with the relevance of the overarching learning outcomes. Students’ evaluations of the coverage of learning outcomes showed a differentiated picture for the MCM. Compared to the regular curriculum track, the coverage in the MCM programme showed substantial improvements in all aspects. Students found themselves to be better prepared for the M2 state examination and the practical year. The students’ overall satisfaction with their decisions to study in the MCM was high.

**Conclusions: **The results of the student evaluation show that a significant improvement in medical education has been achieved at the Charité with the new integrated, outcome-oriented design and the implementation of the MCM. At the same time, ongoing weaknesses have been revealed that serve as a basis for the continued development of the curriculum. This report aims to contribute to the discussion of the future of undergraduate medical education in Germany.

## Zusammenfassung

**Zielsetzung:** Die Einführung der sogenannten „Modellklausel“ gibt den deutschen Fakultäten die Möglichkeit, alternative Konzepte für das Medizinstudium zu erproben. Ziel dieser Projektbeschreibung ist es, die curricularen Kennzeichen des Modellstudiengang Medizin (MSM) der Charité zu skizzieren und das Ergebnis seiner Implementation auf Basis einer semesterübergreifenden Studierendenevaluation zu bewerten.

**Projektbeschreibung: **Der MSM wurde kompetenzbasiert und outcome-orientiert in einem fakultätsweiten Prozess von 2010-2016 geplant und implementiert. Seine Struktur ist charakterisiert durch einen modularen Aufbau, longitudinale Unterrichtsformate und der Integration von Grundlagen und Klinik. Im Wintersemester 2017 wurde eine Studierendenevaluation der MSM-Semester 1-10 durchgeführt. Die Ergebnisse wurden deskriptiv analysiert und bezüglich der Outcome-Erreichung mit Ergebnissen einer Umfrage mit Studierenden des Regelstudiengangs (RSG) von 2016 verglichen.

**Ergebnisse: **An der semesterübergreifenden Studierendenevaluation haben 1047 Studierende teilgenommen (Rücklauf 35%). Ein Großteil bewertet die erreichte curriculare Integration und die longitudinalen Lehrformate positiv. Der Relevanz der übergeordneten Ausbildungsziele wird mehrheitlich zugestimmt. Ihre Vermittlung im MSM zeigt ein differenziertes Bild. Im Vergleich zu Erhebungen zum RSG gibt es in allen Bereichen substanzielle Zugewinne. Verbesserungen finden sich auch beim Vorbereitsein auf die M2-Prüfung und das Praktische Jahr. Die Gesamtzufriedenheit mit der Entscheidung, im MSM zu studieren, ist hoch.

**Schlussfolgerungen: **Die Ergebnisse der Evaluation zeigen, dass aus studentischer Sicht mit der integrierten, outcome-orientierten Konzeption und Implementation des MSM eine deutliche Verbesserung des Medizinstudiengangs an der Charité erreicht wurde. Gleichzeitig werden fortbestehende Defizite aufgezeigt, die als Grundlage für die kontinuierliche Weiterentwicklung des Studiengangs dienen. Dieser Bericht soll zur Diskussion um die Zukunft des Medizinstudiums in Deutschland beitragen.

## 1. Introduction

Medical education has seen fundamental reform in recent decades, both nationally and internationally, with a paradigm shift towards competency-based training [1]. At the core of the competency-based curriculum is the definition of outcomes, which are, on the one hand, derived from the demands of future professional activities and, on the other hand, provide guiding principles for curriculum design [2]. With the introduction of a new “model” clause for reforms in the licensing laws for medical doctors in 1999, numerous German medical faculties have designed and implemented alternative curricula for undergraduate medical education [http://www.gesetze-im-internet.de/_appro_2002/index.html], [3], [4], [5]. Based on experiences with the regular and reformed medical track, the Charité Universitätsmedizin Berlin (Charité) developed and implemented a new modular curriculum of medicine between 2010 and 2016 [6]. The aim was to implement a competency-based and outcome-oriented curriculum as much as possible within the context of formal requirements for undergraduate medical education and existing conditions at the institution. This project report describes the core elements of the MCM and how they were rated based on an across-semester student evaluation.

The paradigm shift towards competency-based education was triggered mainly by current, far-reaching changes in the working lives of physicians: fast medical progress, changing demands of society and patients’ needs as well as new findings from educational research [3], [6]. Traditional, discipline-focused medical tracks are limited in how well they prepare graduates for their working lives as physicians [7], [8]. Criticism has focused on a lack of competency training in the areas of practical clinical skills, physician-patient communication, teamwork skills and scientific skills. Competency-based frameworks, such as the national competency-based learning objectives catalogue (NKLM) [9], which was passed in Germany in 2015, define the overarching outcomes to be achieved by a medical degree programme. These learning outcomes are the basis for the design of outcome-oriented curricula and the further development of existing programmes. An important measure for achieving the defined outcomes is whether students and graduates feel sufficiently prepared for the demands facing them in their working lives as a physician [10], [11]. 

The Charité has a long tradition of reform in medical education. One of the events that triggered this reform was a strike by students to demand the improvement of conditions for studying medicine in the late 1980s [6]. In 1999, this strike led to the introduction of a reformed undergraduate medical curriculum under the leadership of then Dean Dieter Scheffner, which was the first national project to propose an alternative design for the medical track. The reformed curriculum of medicine featured a mainly problem-based learning (PBL) approach and was structured as thematically integrated blocks with intensive early patient contact. Approximately 10% of the entire cohort (n=63 per year) was taught with the reformed curriculum, while 90% continued on the regular curriculum of medicine [12], [13]. In a comparison of the two medical tracks, the students on the reformed curriculum track felt better prepared for their future working lives as physicians and were more satisfied with the programme conditions than those on the traditional curriculum track [7], [14], [15]. Another important result of the reformed medical track was the competency-based formulation of comprehensive learning objectives for the medical degree at the Charité from 2003-2005 (overview in table 1 (Tab. 1)) [https://dsfz.charite.de/]. These objectives were oriented towards international developments, such as Brown’s blueprint for education or the Scottish doctor learning outcomes [16]. In 2006, the joint curriculum committee approved the general learning objectives as the overarching learning outcomes to be achieved by earning a medical degree at the Charité.

In 2010, the Charité faculty decided on the introduction of an integrated, competency-based modular curriculum of medicine (MCM) that would supersede the regular and reformed medicine tracks. A number of factors played a role in this decision; details on this are reported in another article [6]. The above mentioned empirical survey played a key role indicating that students of the reformed medical track considered themselves to be much better prepared for their future work as physicians than students from the regular medical track [7]. 

A six-year, faculty-wide planning and implementation process followed [6]. The MCM was meanwhile fully implemented, and the results of its implementation could be analysed. To do so, a large-scale evaluation was carried out with students in semesters 1 to 10 to rate the curriculum design and the extent to which the intended learning outcomes had been achieved.

This report outlines:

1. the main characteristics of the MCM curricular design and
2. the results of its implementation based on a large-scale student evaluation.

## 2. Project description

### 2.1. Design and implementation of the MCM

The MCM was designed and implemented between 2010 and 2016 as part of a faculty-wide process at the Charité that was based on a new clause in the licensing laws for physicians in an effort to reform medical curricula [6]. The curriculum consists of six years of study with a final-year clerkship (practical year). The overall modular structure design, the learning objectives (outcomes) and the assessment concept were developed by the planning committee for the MCM track (“KEMM”) under the leadership of the dean. A team with interdisciplinary expertise in the fields of basic science, clinical medicine, curriculum development and change management led the MCM project management. Module planning groups developed the programme modules following a structured, 8-step process. The contents of the modules were defined and elaborated by the institutes and clinics represented in the respective modules as well as student representatives [6]. 

The results of the module planning group meetings were reviewed by the curriculum commission with respect to formal aspects and content and, if necessary, were modified. Thereupon the curriculum commission approved for the modules for implementation. 

**Figure 1:**
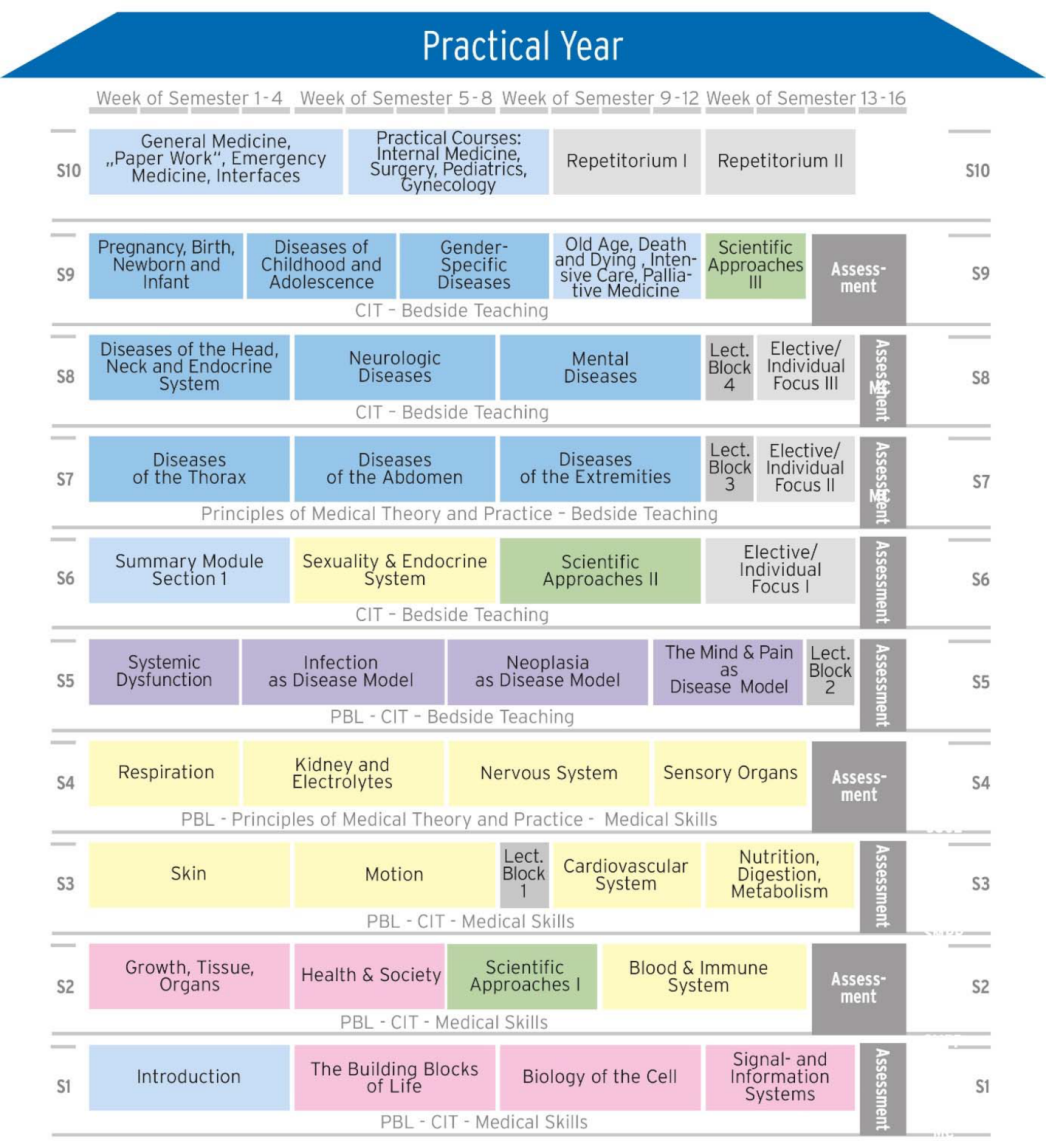
Overview of the modular structure of the modular curriculum of medicine (MCM). The titles and sequences of the modules, the longitudinal teaching formats (“problem-based learning” (PBL), “communication skills and teamwork” (CIT) and the module-supporting lectures) and the examinations over the semester (S) are shown.

The following assessments were planned at different points during the programme: Charité internal examinations at the end of semesters 1-9 and state examinations M2 and M3 after the 5^th^ and 6^th^ years of study. The students would not participate in the M1 examination (“Physikum”). The modular structure of the MCM is illustrated in figure 1 (Fig. 1).

The design of the MCM is characterised by the following core elements:

1. Integration of basic science teaching and clinical, patient-based teaching from the beginning of the programme and basic science teaching up to the 10th semester. Part of this integration is formed by learning spirals with the modules, which build on each other with increasing difficulty in different teaching formats. A further MCM characteristic is an integrated programme with corresponding theoretical and practical examinations.
2. Outcome-oriented curriculum planning based on pre-defined competency-based learning outcomes with the alignment of teaching, learning and assessment based on the learning objectives defined for each teaching session [https://dsfz.charite.de/] (see table 1 (Tab. 1)).
3. Longitudinal teaching formats that interconnect the modules.
  These formats include the following:
  – Bedside teaching with patients, which is divided into general and in-depth patient examination courses (semesters 1-4, 118 teaching units) and bedside teaching (semesters 5-10, 358 teaching units)
  – PBL (semesters 1-5, 240 teaching units)
  – Communication skills and teamwork training (semesters 1-9, 102 teaching units)
  – Scientific skills training in three modules (semesters 2, 6 and 9, together 154 teaching units)
  – Principles of medical theory and practice (semesters 3 and 7, 30 teaching units)
  – Elective modules (semester 6, 60 teaching units; semester 7, 50 teaching units; and semester 8, 50 teaching units) with one topic from a large spectrum of topics offered by the faculty of medicine.

#### 2.2. Across-semester student evaluation

In the winter of 2017/2018, all students in the MCM programme were invited to participate in a large-scale evaluation of the programme. A survey instrument was developed in cooperation with the quality assurance team, the Dieter Scheffner Fachzentrum and student representatives. The questionnaire (5-point Likert scale: 5=completely agree; 1= do not agree) included questions on the achieved integration in the programme, satisfaction with the longitudinal aspects of the programme, the relevance and coverage of the learning outcomes defined for the MCM (with addition of interprofessional training) and the extent to which they felt prepared by the programme for their clerkships, the M2 state examination and the practical year as a result of the programme. Data from students in semesters 1-10 (after the completion of each semester) were included in the analysis. Responses from students in the practical year were not included due to a low response rate. For the items on the relevance and coverage of programme learning outcomes and preparedness, data from students in semesters 7-10 were included in the analysis.

To rank the results on the relevance and coverage of learning outcomes and preparedness, we drew on the results from an earlier 2016 final-year survey. This study, conducted in the summer of 2016, surveyed medical students in the practical year of both the regular and reformed medical tracks. The items in both surveys were identical.

The survey was carried out using the evaluation software EvaSys (Electric Paper Evaluationssysteme GmbH, Lüneburg, Germany). Data were collected in pseudonymised form. The Charité office for data protection approved the study.

Descriptive data were analysed using SPSS 23.0 (IBM SPSS statistics, Armonk, NY, USA). 

## 3. Results

### 3.1. Number of participants

A total of 1,047 of the 2,974 students who were invited participated in the across-semester student evaluation (return rate of 35%; of these respondents, 64% were female students). The students had an average age of 24.5 years (SD: 4.2 years, median: 23 years). A total of 795 students in semesters 1-6 and 252 in semesters 7-10 (return rate of 41% and 25%, respectively) participated in the survey.

A total of 120 students participated in the 2016 final-year survey from the phasing out regular medical track (return rate of 20%; of these respondents, 59% were women). From the MCM track, a total of 64 students participated (return rate of 29%; of these respondents, 69% were women). The students on the regular track were, on average, 28.3 years old (SD: 3.7 years, median: 27 years), and those on the MCM track were, on average, 26.7 years old (SD: 3.5 years, median: 26 years).

The distribution of the sexes in both surveys was similar to that of medical students at the Charité.

#### 3.2. Integration

**Figure 2:**
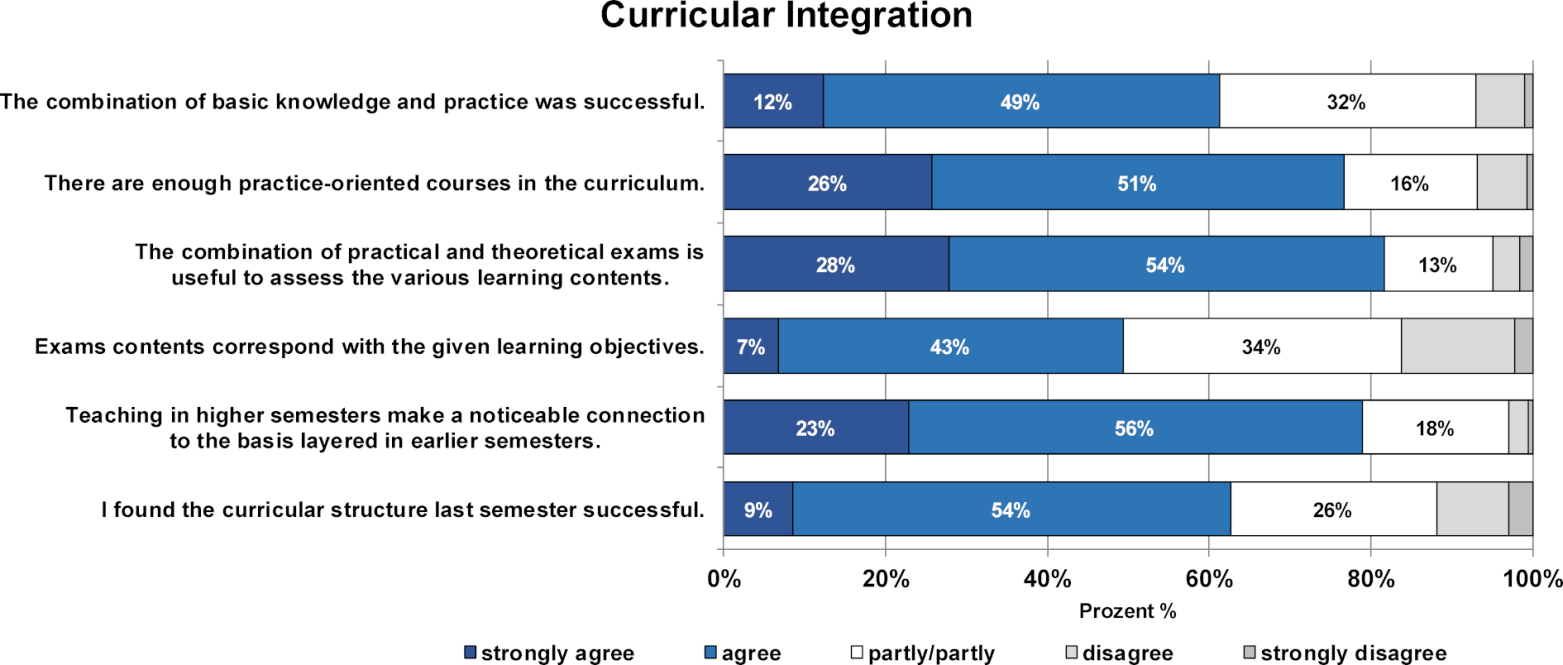
Results of the student evaluations of the characteristics of curricular integration in the MCM. The relative proportions of students who rated the statements on a 5-point Likert scale from "strongly agree" to "strongly disagree" are shown.

The integration of basic and clinical subjects as well as the basic curricular structure received positive ratings from the majority of MCM students (see figure 2 (Fig. 2)). Specifically, the connection of basic science teaching and practical application, the combination of theory and practice in assessments, the alignment of learning objectives and learning spirals across the programme were rated as being (very) successful.

#### 3.3. Longitudinal formats

**Figure 3:**
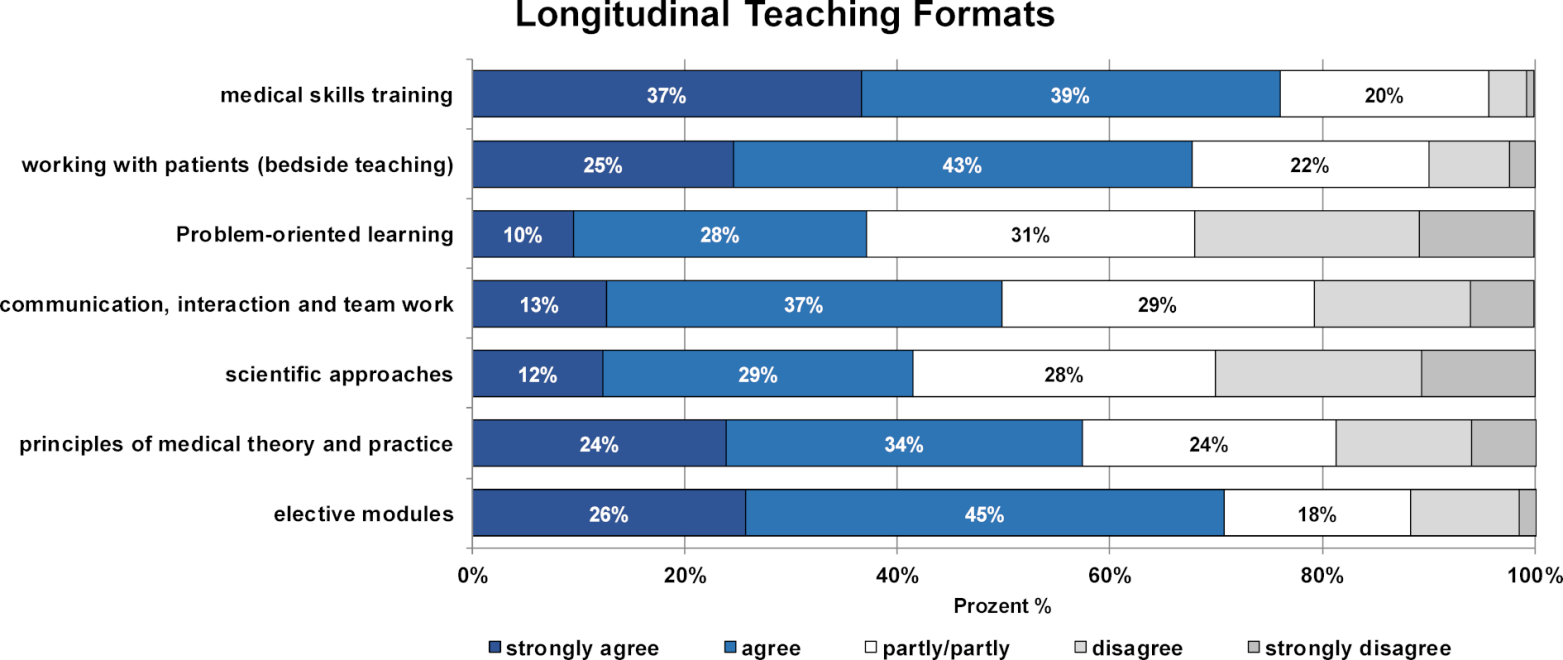
Results of the student evaluation on satisfaction with the longitudinal teaching formats in the Modular Curriculum of Medicine. Shown are the relative proportions of students who rated the statements on a 5-point Likert scale from "strongly agree" to "strongly disagree".

The analysis of student satisfaction with the longitudinal teaching formats on the MCM track yields varied results (see figure 3 (Fig. 3)). The following were rated as highly satisfactory (“completely agree” and “agree”): the patient examination course (76%), bedside teaching (68%), elective modules (71%) and the courses “principles of medical theory and practice” (58%) and “communication skills and teamwork training” (50%). The results indicate less satisfaction with the PBL and scientific skills training formats; although for both of these formats, the proportion of satisfied students (“completely agree” and “agree”) was still higher than that of dissatisfied students (“do not really agree” and “do not agree at all”): PBL, 38% vs. 32%, and scientific skills training, 41% vs. 30%. High satisfaction was reported with the second scientific skills module in which students produce a small scientific work (56% “completely agree” and “agree”).

#### 3.4. Relevance and coverage of overarching learning outcomes

**Figure 4:**
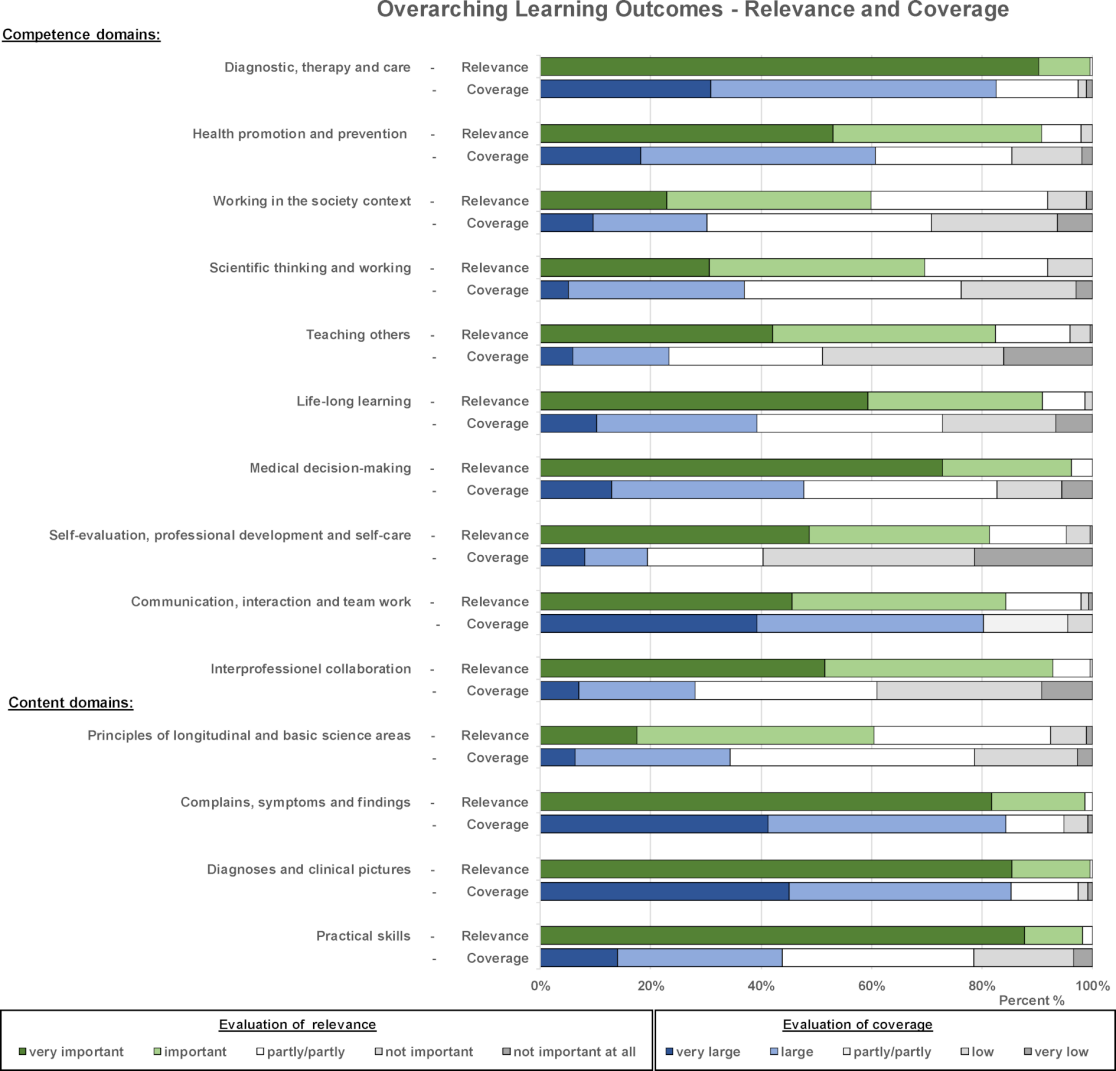
Results of the student evaluations of the relevance of the overarching learning outcomes and their coverage in the MCM. The relative proportions of students rating the statements ("relevant to working as a physician" and "coverage in the MCM") on a 5-point Likert scale from "very important" to "not at all important" and from "very high" to "very low" are shown.

Figure 4 (Fig. 4) shows that all competency and content domains were rated as very important or important for working as a physician by the MCM students. The rating of the coverage of outcomes in their study programme shows a differentiated picture. There was high satisfaction with the coverage in the competency domains “diagnosis, therapy and care” (83%), “health promotion and prevention” (61%) and “communication skills and teamwork” (81%), as well as in the content domains “diagnoses and clinical presentations” (85%) and “complaints, symptoms and findings” (84%). In the other areas, the coverage of teaching in the study programme was rated lower. The lowest rating was recorded for the subjects “teaching others” (26%), “self-evaluation, professional development and self-care” (23%) and “interprofessional collaboration” (29%).

To better interpret the extent to which the overarching learning outcomes had been achieved in the MCM and progress had been made, the results were compared to those of a survey conducted amongst students of both the regular and the modular medicine track during the practical year with respect to the relevance and actual coverage of overarching learning outcomes.

**Figure 5:**
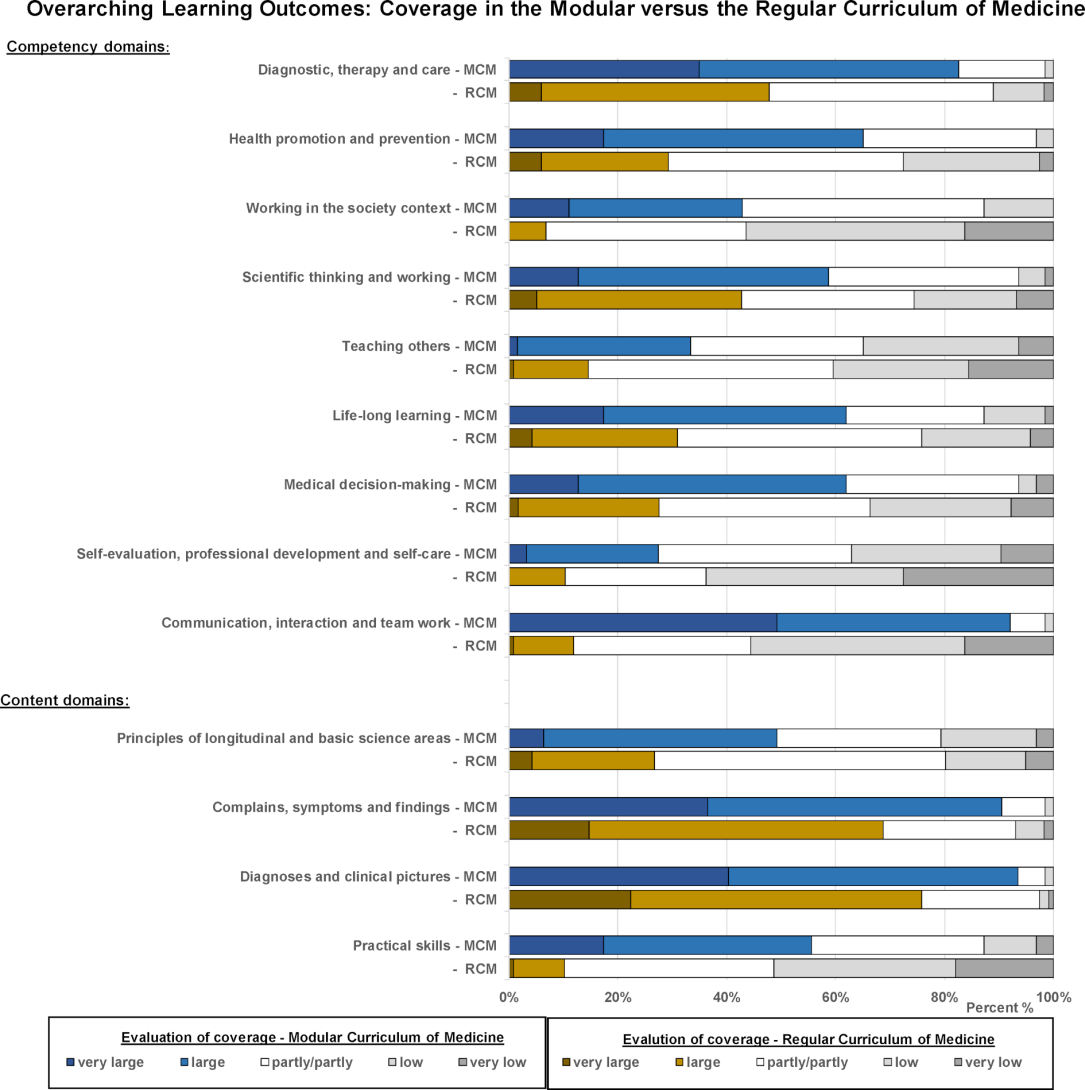
Comparison of the evaluations regarding the coverage of overarching learning outcomes in the modular versus the regular curriculum of medicine (MCM versus RCM). Students evaluated the coverage at the beginning or in the final-year clerk ship of the respective study programme. The relative proportions of student ratings on the degree of coverage on a 5-point Likert scale from "very high" to "very low” are shown.

The results show that there were no fundamental differences in the ratings of the relevance between the two tracks (see table 2 in attachment 1 ). As illustrated in figure 5 (Fig. 5), the coverage of the competency and content domains were much more highly rated by MCM students than by those on the regular track. The most distinct differences existed in the areas of “communication skills and teamwork” and “practical skills”.

#### 3.5. Preparedness and overall satisfaction

Based on the self-evaluations, 64% of MCM students felt well prepared for their final-year clerkships (“completely agree” and “agree”).

**Figure 6:**
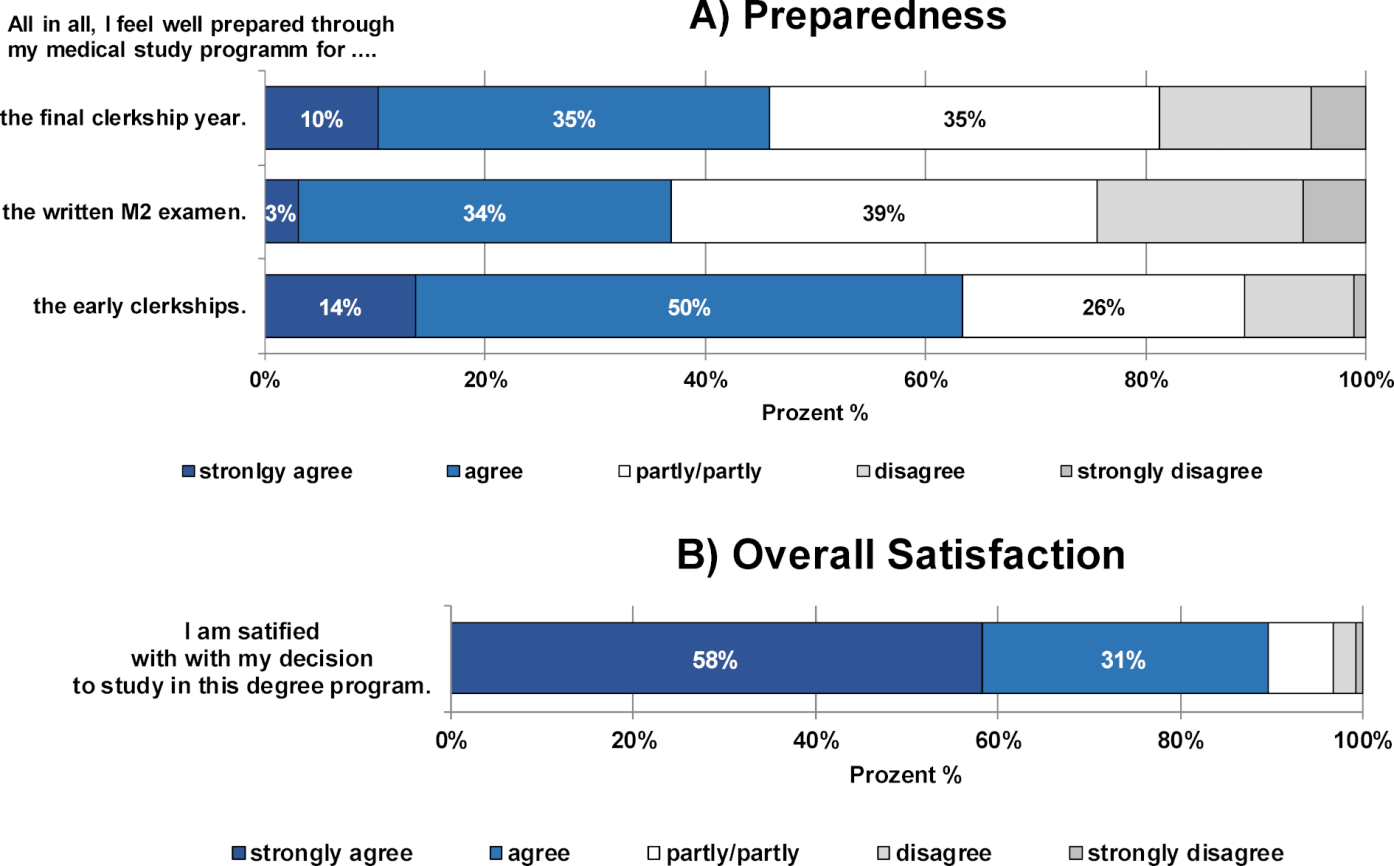
Results of the student evaluations of their preparedness for early clerkships, the written M2 exam and the final-year clerkship (A) as well as their overall satisfaction with the MCM study programme (B). The relative proportions of students who rated the respective statements on a 5-point Likert scale from "strongly agree" to "strongly disagree" are shown.

In the 2016 final-year survey, only 25% of the students on the regular medicine track felt “well prepared for the M2 state examination” and only 19% felt “well prepared for the practical year”. [17] (see figure 6 (Fig. 6)).

In their overall ratings of the MCM, 89% of the students stated that they were satisfied with their decision to study on the MCM track, while only 4% said that they were dissatisfied.

## 4. Discussion

The introduction of a reform clause in German approbation laws for physicians set two goals. On the one hand, it defined the legal framework within which medical faculties in Germany can develop and implement alternative designs for the undergraduate medical curriculum [http://www.gesetze-im-internet.de/_appro_2002/index.html]. On the other hand, it included an obligation to evaluate these new concepts and to contribute to the further development of the approbation law for physicians. The recently published report by the scientific board (Wissenschaftsrat) on the further development of the medical track and the master medical plan for 2020 highlighted similar directions [15], [18]. With this project report on the MCM at the Charité and the large-scale student evaluation, we aim to contribute to the discussion on the future of undergraduate medical education in Germany.

In summary, the results of the student evaluation show that the majority of students were satisfied with their decision to study in the MCM programme. The levels of satisfaction with the study choice are higher than the reported already good general approval ratings in other national and european medical studies [19], [20], [21]. We found especially high levels of satisfaction in numerous aspects of integration as a core feature of this curriculum, as well as for most of the longitudinal teaching formats. MCM students rated the coverage of the overarching learning outcomes substantially higher than students did on the regular medicine track. In addition, MCM students gave higher self-evaluations of their preparedness for practical clerkships, such as in the practical year. Meanwhile, the evaluation reveals numerous areas with potential for improvement.

With respect to the challenges involved in such far-reaching curricular reform as the MCM, we can conclude from this evaluation that from the students’ points of view, many aspects of this reform at the Charité appear to have been successful. The following section of the article discusses the results of the evaluation as an analysis of strengths and weaknesses. As strengths, two areas are emphasized:

1. The first area includes the longitudinal teaching formats for “medical skills course” and “bedside teaching” and the teaching coverage in the MCM programme in the areas of “diagnosis, therapy and medical care”, “complains, symptoms and findings” and “diagnosis and clinical pictures”. This strength reflects the fact that in the MCM curriculum, “teaching with patients” is a central component of the study programme and the development of competencies by students. Teaching from the first semester onwards is based on professional activities, which grow in complexity over the course of study. Meanwhile, expectations regarding the necessary level of supervision while carrying out these activities decrease. This longitudinal teaching format is based on the concept of entrustable professional activities, which has been increasingly recognised as a helpful tool for competency-based training in medical education [22], [23], [http://www.profilesmed.ch].
2. The second strength is the longitudinal teaching format for communication skills and teamwork in combination with the high ratings of the importance of this competency area for medical practice and its integration in the modular curriculum. The strengths of the communication curriculum in the MCM are, amongst others,
  – the close connection between the taught subjects in physician-patient communication and teamwork with the topics of the respective modules,
  – the close connection to bedside teaching,
  – the recruitment of standardized actors as patients and
  – the integration of the subject into practical assessments.

With regard to weaknesses, we focus on two areas. The first weakness is that improvement in the area of medical decision-making was weaker than expected. A likely reason for this lack of improvement is that bedside teaching was not sufficiently well structured and cardinal symptoms were still insufficiently addressed in the MCM. Two interventions to improve this situation have therefore already been introduced:

1. An interdisciplinary working group commissioned by the MCM curriculum committee developed a plan for the structured teaching of “medical decision-making” for bedside teaching courses. This plan is currently being implemented.
2. The current MCM curriculum was mapped to compare the already included cardinal symptoms and the overall learning outcomes (the “complaints, symptoms and findings” content area and the NKLM, chapter 20, reasons for consultations) [https://dsfz.charite.de/], [http://www.nklm.de].

The curriculum committee and the module planning groups will build on these expected results to improve the inclusion of more cardinal symptoms and their differential diagnoses in the MCM.

A second area for improvement is that there is currently insufficient inclusion of inter-professional education (IPE) in the MCM. This situation has also been reported in other medical faculties [24]. Supported by the Robert Bosch Foundation, teaching courses have recently been introduced at the Charité with students from other health professions. These teaching units have been introduced in both regular sessions and student tutorials [24], [25]. Building on these pilot project experiences, a Charité-wide network for IPE has recently been launched. This project is closely connected to local, national and international IPE networks. Cross-curriculum and professional overall learning objectives for IPE and a longitudinal curriculum for IPE in the modular medical track are currently being developed at the Charité.

Altogether, the evaluation results presented here have led to concrete improvement measures for the MCM, which underlines the significance and validity that the faculty attribute to the results. The above-described improvement measures were a key component of the application to the Berlin senate for the extension of the MCM programme. All in all, the introduction of the new MCM at the Charité has led to a culture in which curricular reform is regarded as a continual, faculty-wide process.

This report has limitations in that it is based on the experiences of one medical faculty and is not necessarily transferable to other faculties. In addition, the study reform programme was evaluated from a student perspective. Feedback from university lecturers, other teachers and staff was not taken into account in this project report. Study periods and exam results should also be included for a more complete picture. In addition, the return rates for the evaluations, which were based on voluntary participation, were comparatively low, which raises the question of representability [11]. However, evaluations based on mandatory participation carry the risk of generating improper responses. The results of this report are based on a high absolute number of participants and are consistent between surveys. In the 2016 final-year survey, the students in the traditional study programme were older than those in MCM programme. Their assessments of how well the respective study programmes covered the overarching learning outcomes for medical studies at the Charité should not have been influenced to an appreciable extent.

## 5. Conclusions

Building on the experiences of the reformed medical track, the Charité has introduced a competency-based, outcome-oriented and integrated practice model with the MCM programme. Student evaluations showed substantial improvements in comparison to the previous regular curriculum of medicine at the same institution. The implementation of the modular medical track has led to a culture of continual improvement in medical education at the Charité.

## Acknowledgements

The authors would like to thank all students who participated in this survey as well as the student representatives who developed the survey and recruited participants. Special thanks go to the whole development team of the reformed medical track under the leadership of Dieter Scheffner for their invaluable, pioneering work. We would like to thank our colleagues Johanna Balz, Josefin Bosch, Martina Klau-Fadke, Ylva Holzhausen, Rita Kraft, Martin Krebber and Peter Kube, without whom this survey and the analysis would not have been possible. Our thanks also go to the colleagues of the steering group for modular degree planning: Peter Arends, Olaf Ahlers, Irene Brunk, Antje Degel, Jakob Hein, Julia Karner, Judith Mörschner, Jörg Pelz, Charles Christoph Röhr, Konstanze Vogt, and Jishun Zhu. We would also like to thank several student members of the project steering committee: Sara Katzenstein, Lennart Milles, Andia Mirbagheri, Agata Mossakowski, Oliver Mossakowski, and Steffen Willun. 

## Competing interests

The authors declare that they have no competing interests. 

## Supplementary Material

Table 2

## Figures and Tables

**Table 1:**
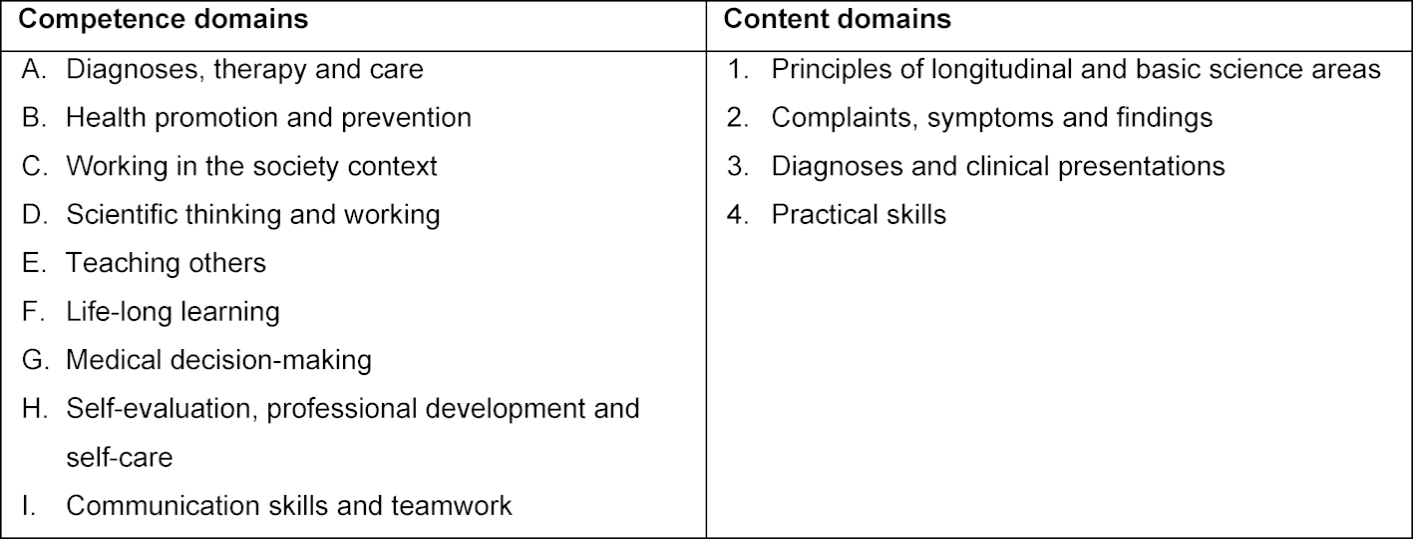
Overview of the competence and content domains at the Charité, which served as a framework for the outcome-oriented curriculum planning of the modular curriculum of medicine (MCM).

## References

[1] Frank JR, Snell LS, Cate OT, Holmboe ES, Carraccio C, Swing SR, Harris P, Glasgow NJ, Campbell C, Dath D, Hareden RM, Iobst W, Long DM, Mungroo R, Richardson DL, Sherbion J, Silver I, Taber S, Talbot M, Harris KA (2010). Competency-based medical education: theory to practice. Med Teach.

[2] Carraccio C, Englander R, Van Melle E, ten Cate O, Lockyer J, Chan MK, Frank JR, Snell LS, International Competency-Based Maciel Education Collaborators (2016). Advancing Competency-Based Medical Education: A Charter for Clinician-Educators. Acad Med.

[3] Guse AH, Kuhlmey A (2018). Modellstudiengänge in der Medizin: Lehrinnovationen am Beispiel der Studiengänge in Hamburg und Berlin. Bundesgesundheitsbl Gesundheitsforsch Gesundheitsschutz.

[4] Fürstenberg S, Schick K, Deppermann J, Prediger S, Berberat PO, Kadmon M, Harendza S (2017). Competencies for first year residents - physicians' views from medical schools with different undergraduate curricula. BMC Med Educ.

[5] Gehlhar K, Wuller A, Lieverscheidt H, Fischer MR, Schäfer T (2010). Is a PBL curriculum a better nutrient medium for student-generated learning issues than a PBL island?. Adv Health Sci Educ Theory Pract.

[6] Maaz A, Hitzblech T, Arends P, Degel A, Ludwig S, Mossakowski A, Mothes R, Breckwoldt J, Peters H (2018). Moving a mountain: Practical insights into mastering a major curriculum reform at a large European medical university. Med Teach.

[7] Dettmer S, Kuhlmey A, Schwarz F, Angerer P (2010). Studienzufriedenheit und berufliche Zukunftsplanung von Medizinstudierenden - ein Vergleich zweier Ausbildungskonzepte. Arbeitsbedingungen und Befinden von Ärztinnen und Ärzten Befunde und Interventionen.

[8] Schwarzer A, Fabian G (2012). Medizinerreport 2012 - Berufsstart und Berufsverlauf von Humanmedizinerinnen und Humanmedizinern.

[9] Fischer MR, Bauer D, Mohn K (2015). Finally finished! National Competence Based Catalogues of Learning Objectives for Undergraduate Medical Education (NKLM) and Dental Education (NKLZ) ready for trial. GMS Z Med Ausbild.

[10] Burford B, Vance G (2014). When I say ... preparedness. Med Educ.

[11] Bosch J, Maaz A, Hitzblech T, Holzhausen Y, Peters H (2017). Medical students' preparedness for professional activities in early clerkships. BMC Med Educ.

[12] Kiessling C, Schubert B, Scheffner D, Burger W (2004). First year medical students' perceptions of stress and support: a comparison between reformed and traditional track curricula. Med Educ.

[13] Müller B, Robert Bosch Stiftung (2011). Entwicklung und Bewertung der Ansätze zur Reform der Medizinerausbildung 1989 bis 2009. Ausbildung für die Gesundheitsversorgung von morgen.

[14] Burger W, Dudenhausen J (2003). Reform des Medizinstudiums. Positive Erfahrungen an der Charité Berlin. Dtsch Ärztebl.

[15] Wissenschaftsrat (2014). Empfehlungen zur Weiterentwicklung des Medizinstudiums in Deutschland auf Grundlage einer Bestandsaufnahme der humanmedizinischen Modellstudiengänge.

[16] Holzhausen Y, Maaz A, Renz A, Peters H (2019). Development of Entrustable Professional Activities for entry into residency at the Charité. GMS J Med Educ.

[17] Ludwig S, Dettmer S, Wurl W, Seeland U, Maaz A, Peters H Evaluation of curricular relevance and actual integration of sex/gender and cultural competencies by final year medical students: effects in student diversity subgroups and by curriculum.. GMS J Med Educ.

[18] Bundesministerium für Bildung und Forschung (2017). Masterplan 2020.

[19] Baschera D, Westermann L, Isenegger P, Zellweger R (2015). tudienzufriedenheit und Lebensstil von Medizinstudenten im deutschsprachigen Raum. Dtsch Med Wochenschr.

[20] Pike G (1991). The effects of background, coursework, and involvement on students' grades and satisfaction. Res High Educ.

[21] De Sterke A (2018). Einflussfaktoren auf die Studienzufriedenheit und berufliche Lebensplanung von Medizinstudierenden der Charité - Universitätsmedizin Berlin (Thesis).

[22] Holzhausen Y, Maaz A, Renz A, Bosch J, Peters H (2018). How to define core entrustable professional activities for entry into residency?. BMC Med Educ.

[23] Ten Cate O, Graafmans L, Posthumus I, Welink L, van Dijk M (2018). The EPA-based Utrecht undergraduate clinical curriculum: Development and implementation. Med Teach.

[24] Bohrer A, Heinze C, Hoppner H, Behrend R, Czakert J, Hitzblech T, Kaufmann I, Maaz A, Räbiger J, Peters H (2016). Berlin in Motion: Interprofessional teaching and learning for students in the fields of medicine, occupational therapy, physiotherapy and nursing (INTER-M-E-P-P). GMS J Med Educ.

[25] Reichel K, Dietsche S, Hölzer H, Ewers M (2016). Interprofessional peer-assisted learning as a low-threshold course for joint learning: Evaluation results of the interTUT Project. GMS J Med Educ.

