# Supplementary material for: The modular curriculum of medicine at the Charité Berlin – a project report based on an across-semester student evaluation
Source: GMS J Med Educ. 2019 Oct 15;36(5):Doc54. doi: 10.3205/zma001262 (PMC6883251; doi:10.3205/zma001262)
Supplement: Table 2 [file JME-35-54-s-001.pdf]

Table 2**:** Ratings on the relevance of Outcomes compared between students from the Modular and Regular Curriculum of Medicine. Students evaluated the relevance at the beginning or in the practical year of the respective study program. The relative proportions of student statements assessing relevance for working as a physician on a five-point Likert scale from "very important" to "not at all important" are shown.

| | | | | | | | Sum |
|---|---|---|---|---|---|---|---|
| | Study programme | very important | important | partly/partly | less important | not important at all | Very important and important |
| **Competency domain** | | | | | | | |
| Diagnostic, therapy and care | Modular Curriculum | 60,3% | 1,6% | 1,6% | 1,6% | 34,9% | 61,9% |
| | Regular Curriculum | 76,1% | 2,6% | 0,0% | 6,0% | 15,4% | 78,6% |
| Health promotion and prevention | Modular Curriculum | 39,3% | 45,9% | 9,8% | 4,9% | 0,0% | 85,2% |
| | Regular Curriculum | 44,4% | 47,9% | 6,8% | 0,9% | 0,0% | 92,3% |
| Working in the society context | Modular Curriculum | 23,8% | 46,0% | 19,0% | 11,1% | 0,0% | 69,8% |
| | Regular Curriculum | 25,6% | 49,6% | 22,2% | 2,6% | 0,0% | 75,2% |
| Scientific thinking and working | Modular Curriculum | 23,8% | 50,8% | 23,8% | 1,6% | 0,0% | 74,6% |
| | Regular Curriculum | 21,4% | 43,6% | 27,4% | 6,8% | 0,9% | 65,0% |
| Teaching others | Modular Curriculum | 42,9% | 50,8% | 3,2% | 3,2% | 0,0% | 93,7% |
| | Regular Curriculum | 46,6% | 44,0% | 8,6% | 0,9% | 0,0% | 90,5% |
| Life-long learning | Modular Curriculum | 65,1% | 31,7% | 1,6% | 1,6% | 0,0% | 96,8% |
| | Regular Curriculum | 70,1% | 25,6% | 4,3% | 0,0% | 0,0% | 95,7% |
| Medical decision-making | Modular Curriculum | 61,9% | 34,9% | 3,2% | 0,0% | 0,0% | 96,8% |
| | Regular Curriculum | 63,2% | 31,6% | 5,1% | 0,0% | 0,0% | 94,9% |
| Self-evaluation, professional development and self-care | Modular Curriculum | 42,9% | 42,9% | 9,5% | 4,8% | 0,0% | 85,7% |
| | Regular Curriculum | 41,4% | 50,9% | 6,9% | 0,9% | 0,0% | 92,2% |
| Communication, interaction and team work | Modular Curriculum | 47,6% | 39,7% | 9,5% | 1,6% | 1,6% | 87,3% |
| | Regular Curriculum | 64,1% | 32,5% | 3,4% | 0,0% | 0,0% | 96,6% |
| **Content domain** | | | | | | | |
| Principles of longitudinal and basic science areas | Modular Curriculum | 14,3% | 28,6% | 47,6% | 7,9% | 1,6% | 42,9% |
| | Regular Curriculum | 11,2% | 38,8% | 41,4% | 6,9% | 1,7% | 50,0% |
| Complains, symptoms and findings | Modular Curriculum | 81,0% | 17,5% | 1,6% | 0,0% | 0,0% | 98,4% |
| | Regular Curriculum | 69,8% | 26,7% | 3,4% | 0,0% | 0,0% | 96,6% |
| Diagnoses and clinical pictures | Modular Curriculum | 85,7% | 11,1% | 3,2% | 0,0% | 0,0% | 96,8% |
| | Regular Curriculum | 63,8% | 32,8% | 3,4% | 0,0% | 0,0% | 96,6% |
| Practical skills | Modular Curriculum | 88,9% | 7,9% | 3,2% | 0,0% | 0,0% | 96,8% |
| | Regular Curriculum | 80,2% | 19,0% | 0,0% | 0,9% | 0,0% | 99,1% |
| Gender-sensitive approach to patients | Modular Curriculum | 14,5% | 46,8% | 24,2% | 11,3% | 3,2% | 61,3% |
| | Regular Curriculum | 23,9% | 38,5% | 29,1% | 6,0% | 2,6% | 62,4% |
| Culture-sensitive approach to patients | Modular Curriculum | 23,8% | 52,4% | 14,3% | 7,9% | 1,6% | 76,2% |
| | Regular Curriculum | 29,6% | 40,9% | 23,5% | 2,6% | 3,5% | 70,4% |
